# Mobile colposcopy by trained nurses in a cervical cancer screening programme at Battor, Ghana

**DOI:** 10.4314/gmj.v56i3.3

**Published:** 2022-09

**Authors:** Kofi Effah, Mawusi C Wormenor, Ethel Tekpor, Joseph E Amuah, Hayford B Atuguba, Essel N O Mensah, Ewoenam S Badzi, Stephen Danyo, Dominic Agyiri, Gifty B Klutsey, Patrick K Akakpo

**Affiliations:** 1 Catholic Hospital, Battor, Ghana; 2 School of Epidemiology and Public Health, Faculty of Medicine, University of Ottawa, Ottawa, Ontario, Canada; 3 Lotus Medical Group, Kumasi, Ghana; 4 Department of Public Health, The Salvation Army Hospital, Agona-Duakwa, Ghana; 5 Department of Pathology, School of Medical Sciences, University of Cape Coast, Cape Coast Teaching Hospital, Cape Coast, Ghana

**Keywords:** Colposcopy, nurses, enhanced, cervix, cancer, screening

## Abstract

**Objectives:**

Cervical precancer screening programs are difficult to establish in low resource settings partly because of a lack of human resource. Our aiming was to overcome this challenge. We hypothesized that this could be done through task shifting to trained nurses.

**Design:**

Descriptive retrospective cross-sectional review.

**Setting:**

Training was at the Cervical Cancer Prevention and Training Center (CCPTC) and screening was carried out at the clinic and at outreaches / peripheral facilities.

**Participants:**

All women who reported to the clinic for screening or were recruited during outreaches

**Interventions:**

All 4 nurses were trained for at least 2weeks (module 1). A total of 904 women were screened by the trained nurses using the EVA system. Quality assurance was ensured.

**Main outcome measures:**

Primary screening and follow-up were carried out by the trained nurses with quality assured through image sharing and meetings with peers and experienced gynaecologists.

**Results:**

828 women had primary screening and 76 had follow-up screening. 739 (89.3%) were screened at the clinic and 89 (10.7%) at outreaches/peripheral facilities. Of all screened, 130 (14.5%) had cervical lesions, and 25 (2.8%) were treated, 12 (48.0%) by Loop Electrosurgical Excision Procedure (LEEP) performed by a gynaecologist, 11 (44.0%) with thermal coagulation by trained nurses except one, and 2 (8.0%) with cryotherapy by trained nurses.

**Conclusion:**

We demonstrate the utility of a model where nurses trained in basic colposcopy can be used to successfully implement a cervical precancer screening and treatment program in low-resource settings.

**Funding:**

None indicated

## Introduction

In current practice, colposcopy is the cornerstone for properly identifying the transformation zone, grading identified lesions and biopsying high-grade cervical intraepithelial neoplasia.^1^ Its operation and diagnostic yield depend on the training and expertise of the operator and the operational setting.^2^ Mobile and digital colposcopy (the so-called “smartscopy”^3^) has several advantages that make it easier and more prudent to use in most settings compared to its optical counterpart or traditional colposcopes. Recently, high-resolution images taken with high-definition cameras of mobile colposcopes are much preferred because they improve the detection of significant lesions and enable images to be shared between supervising colposcopists and their trainees (usually junior or middle-cadre staff).^4^,^5^ Despite its great exactitude in the triage of patients shown to have abnormal cytologic findings, its availability in the developing world somewhat limits its use in these contexts where it is most needed, primarily due to its high cost.^1^ That said, it is still cheaper than a fixed colposcope, and its ease of use means that if available, it can be used in remote settings where hitherto, traditional coloscopy was impossible.

With the focus nowadays on meeting the need for simple yet cost-effective methods of diagnosing cervical lesions among women, interest in mobile colposcopy has increased.

Smartphones, mobile networks, and their clinical applications are almost ubiquitous. There have been reports of the application of high-definition cameras on smartphones in diagnostic pathology as part of telemedicine setups, as well as the use of social media applications like WhatsApp Messenger in seeking the second opinion among clinicians, particularly using the “group chat” feature.^6^,^7^ Among the available mobile colposcopes, the Enhanced Visual Assessment (EVA) system (MobileODT Ltd., Tel Aviv, Israel) was developed to, in addition to many other things, enhance visual inspection with acetic acid (VIA) procedures in resource-limited settings.^8^,^9^,^10^ The system incorporates a smartphone application into a mobile colposcope, allowing for image storage and annotation.^8^ Further, using such mobile colposcopy protocols for triaging is expected to increase their effectiveness and reach, as they can be used in more remote settings with basic infrastructure.

In low-middle income countries, there is an increasing need to create additional avenues for cancer care due to the rarity of specialist services and other resource constraints.^11^ In these parts, nurses act as principal actors (in a multidisciplinary team) in providing primary and secondary preventive services for a few cancers, including carcinoma of the cervix. They enhance diagnostic and treatment coordination and provide culturally appropriate patient education tailored to patients' education levels and socioeconomic backgrounds.^12^ While playing supportive roles in early diagnosis and management, they also support women with abnormal screening results via post-screening education and referrals.

Studies from the developed world have indicated that trained nurses can perform oncologic screening procedures with non-inferior ratings regarding patient satisfaction, effectiveness, and safety^13^–^15^ compared with specialists and senior specialists. The findings of these studies imply that when appropriately trained, nurses in resource-limited settings can take up this level of care through task-shifting (via mobile colposcopy, in this case). Despite this, barriers to expanding nurses' scope of practice in the oncology field in resource-limited countries persist. Only a few sites are available to train nurses in the areas where their services are needed. Again, most nurses need to be sponsored to benefit from such training, even if the training sites are available. In addition, once trained, these nurses need to be resourced to perform their new roles, and this may not be possible due to a lack of funding. The CCPTC is one of a handful of sites in Ghana that runs a dedicated program for training nurses to carry out cervical cancer screening and treatment of pre-cancer. Students are supported through grants from individuals and organisations and graduates are also resourced and supported to set up screening centres where they come from. Through this initiative, many screening centres have been set up across the country.

The value of nurses in cervical carcinoma control (screening and treating precancers) has been demonstrated in other low-resource settings, such as India.^16^ The approach used by the Cervical Cancer Prevention and Training Center (CCPTC), Battor, Ghana, is a high yield, low-cost one, in which a team of nurses and middle-cadre staff co-diagnose and co-manage patients with specialists who act as mentors and provide feedback, guidance, and didactical training. With this approach, these nurses become equipped with the necessary skills, self-confidence, independence, and knowledge base to manage complex conditions at the community level^12^,^15^, and act as trainers of trainers by mentoring nurses from other sponsored districts and communities to set up cervical cancer screening facilities. They help reduce travel time, wait time, costs, and complications associated with these delays. In this form of “telementoring”, unlike the traditional approach to telemedicine, each nurse retains their duty of care to patients as their skills and independence develop instead of referring patients, many of whom do not follow up with such referrals.

By spotlighting how nurses contribute to the provision of oncologic services in resource-limited settings, we hope to address issues faced by this workforce and provide suggestions as to how they can be subverted and indicate the need to strengthen their expertise and expand their current role. Thus, the present paper aims to share our ongoing experience and algorithms with using nursing staff as providers of cervical cancer screening and treatment services at the CCPTC, Battor, situated in a secondary health care setting in Ghana, and call into action the need to provide education and oncologic training to nurses in similar settings.

## Methods

### Setting and study design

Catholic Hospital, Battor serves as the district hospital in the North Tongu District, one of the 18 administrative districts/Municipalities in the Volta Region. The hospital serves a rural population but receives many clients from nearby urban settings. The Cervical Cancer Prevention and Training Centre (CCPTC) was established in 2017 and, apart from offering cervical cancer prevention services, trains health workers (nurses, midwives, physician assistants and medical doctors) to offer cervical cancer prevention services.

**Figure 1:**
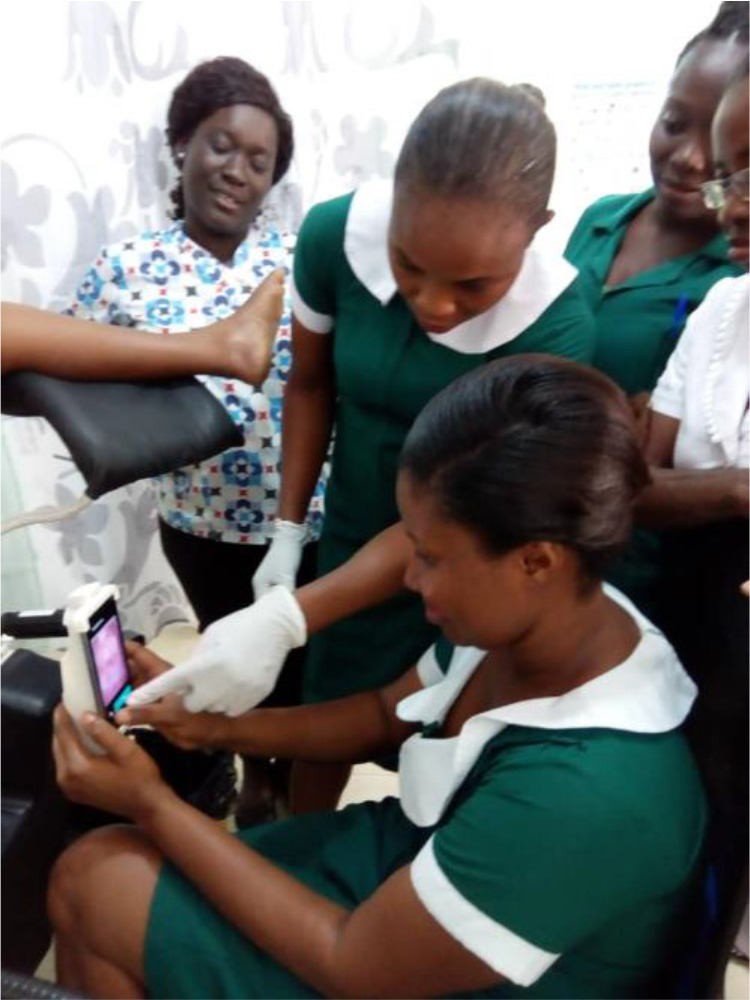
Nurses and midwives being trained to use the mobile colposcope at the CCPTC, Battor

**Figure 2:**
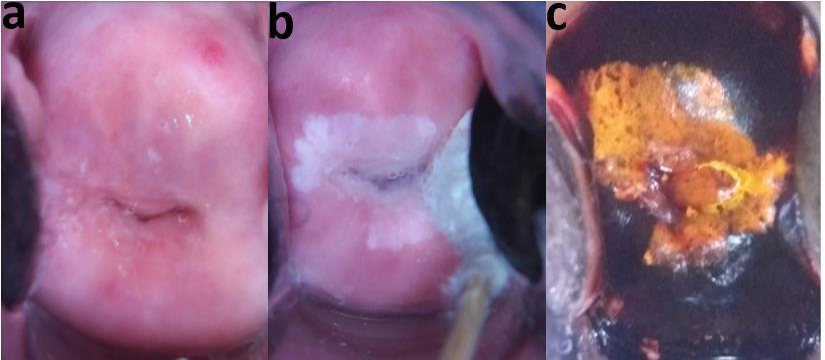
39 years, para 4, careHPV Positive. Colposcopy with EVA system by a nurse: major change - dense aceto-whitening (‘inner border sign’ - ‘lesion within a lesion’), transformation zone type 3. Pap smear: High-grade Squamous Intraepithelial Lesion (HSIL). Treatment by LEEP. Histopathology: CIN 3. a. Before acetic acid. b. After acetic acid. c. After Lugol's iodine

**Figure 3:**
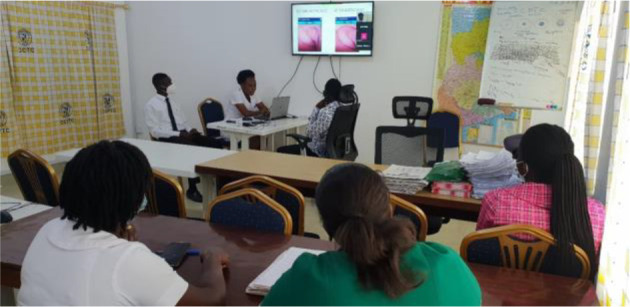
Quality assurance meeting

**Figure 4:**
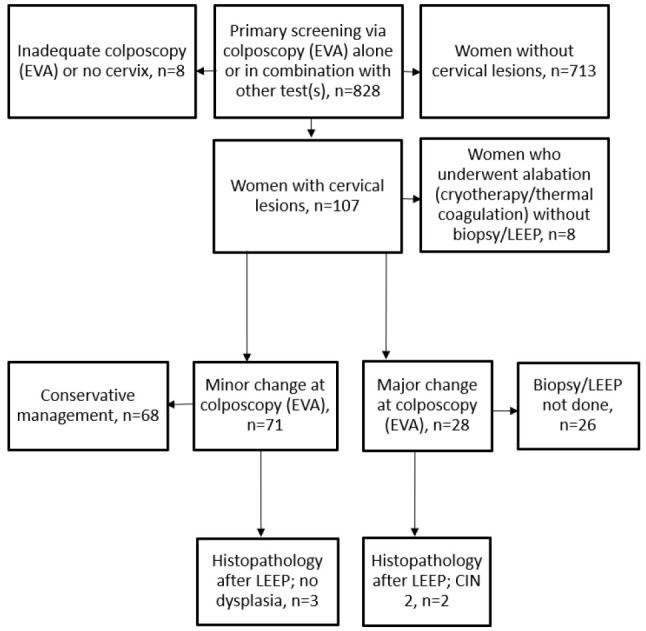
Flow chart of primary screening using mobile colposcopy (EVA) by nurses

**Figure 5:**
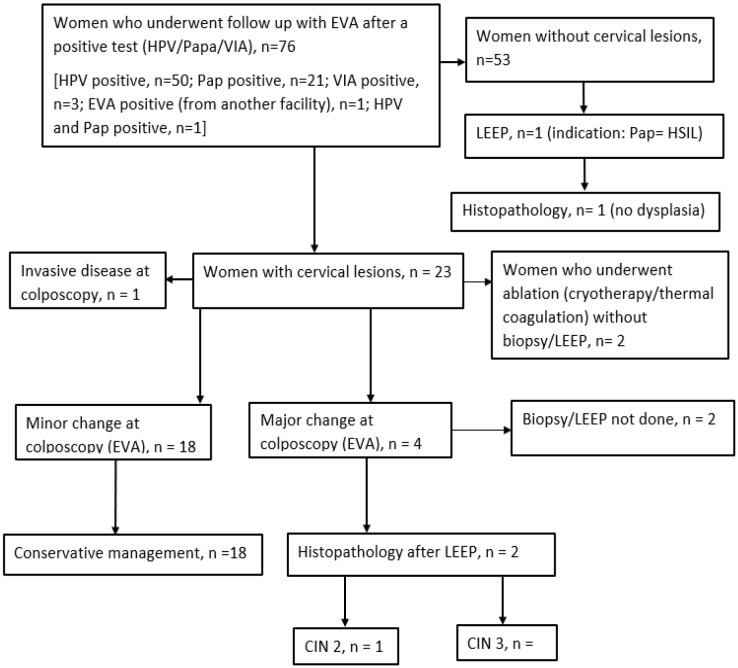
Flow chart of follow-up screening using mobile colposcopy (EVA) by nurses.

This study is a descriptive retrospective cross-sectional review of 904 women screened and followed up by four trained nurses using a mobile colposcope, the Enhanced Visual Assessment (EVA) system (MobileODT, Tel Aviv, Israel) (figure 1 & 2). Nurses share anonymised images (all positive cases) with a specialist via WhatsApp©. Though possible, a cloud storage system was not used to reduce cost. The use of algorithms ensures that care is standardised (figure 4&5). There are also monthly quality assurance meetings where all the cases for the previous month are discussed (figure 3). Images of screen positives are shown and discussed at the monthly quality assurance meetings. There has not been any objective assessment of the quality assurance yet, but very few changes are made to the management plans of the nurses because of the avenues to discuss cases physically with a specialist/gynaecologist or through sharing anonymised images on smartphones.

### Training of nurses and work after training

**Figure 6:**
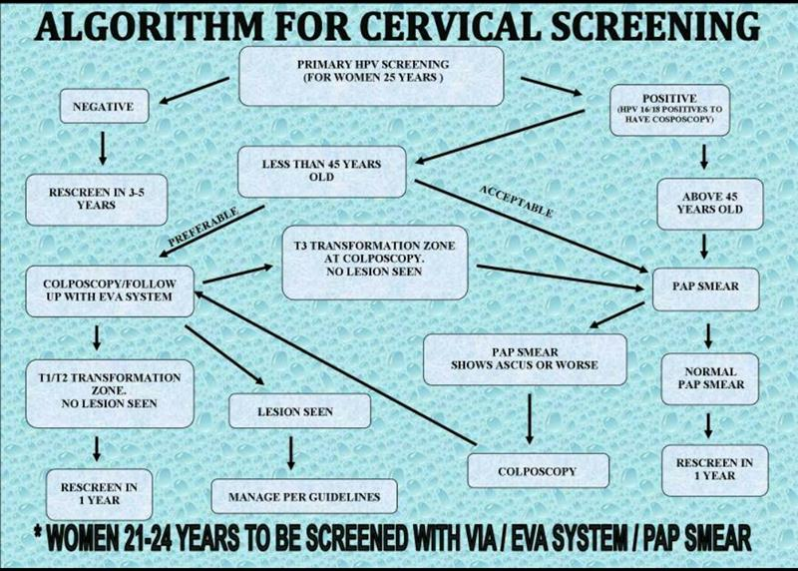
Algorithm for cervical cancer screening developed by the CCPTC, Battor.

**Figure 7:**
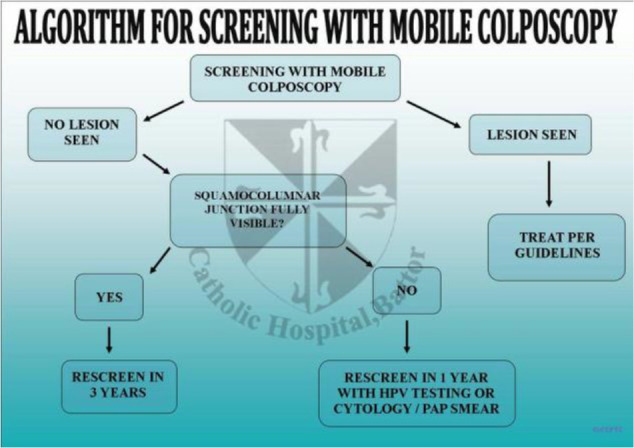
Algorithm for cervical cancer screening using the mobile colposcope.

**Figure 8:**
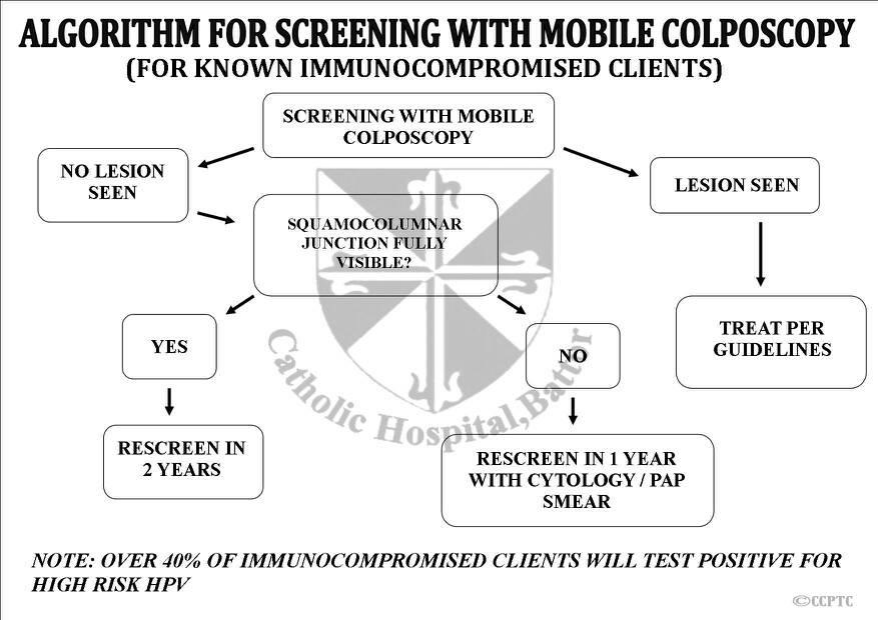
Algorithm for cervical cancer screening with the mobile colposcope for known HIV-positive patients.

All 4 nurses involved had been trained in at least Module 1 of the cervical cancer course offered at the CCPTC (Appendix 1). This lasts for two weeks, and the core competencies include understanding the development of cervical precancer and cancer and how to perform Visual Inspection with Acetic acid and Basic Colposcopy. Module 1 equips trainees with skills to set up a cervical cancer screening unit using VIA and HPV DNA testing and cytology if these are available to trainees at their centres. Trainees also learn basic colposcopy and can use (mobile) colposcopes. After training, the nurses followed the algorithms developed by the CCPTC in screening women in the clinic in Battor and on outreaches (Figures 6, 7, 8). The nurses continued to develop themselves after training through discussions in the clinic, discussing anonymised images remotely with gynaecologists and monthly Quality Assurance meetings with a gynaecologist in attendance who reviewed the management of cases seen.

Module 2 equips trainees with skills to treat premalignant lesions of the cervix using ablation (cryotherapy/thermal coagulation). Nurses are taught to report their colposcopy findings using the 2011 International Federation for Cervical Pathology and Colposcopy nomenclature. 17 The core competencies expected of trainees of the program are shown in Appendix 2.

### Cervical screening for clients at CCPTC, Battor

HPV DNA testing as primary screening for cervical pre-cancers began in Catholic Hospital, Battor, in June 2016. Nurses trained in Basic Colposcopy started performing colposcopy using the EVA system in September 2016.

Clients pay for screening out of pocket, and all data on clients and their screening status is captured and stored securely in databases managed by the CCPTC.

The Mobile Colposcope (EVA System) is used for the following in Battor:

1. Primary screening, together with VIA, PAP smear and HPV DNA testing (using careHPV, GeneXpert and AmpFire systems)
2. Follow-up of screen positives after the primary screening with HPV DNA testing and PAP smear as described in (1)

Using a mobile colposcope (EVA System) allows images to be captured, stored, magnified and shared to discuss case findings. The images are all anonymised and are shareable via messaging Apps like WhatsApp©. This enables the nurses to consult specialists worldwide for their input, even in real-time. All women screened met the eligibility criteria for screening with the EVA, as mentioned above. These indications also fit into the algorithm used for screening at the CCPTC. A client may be screened using multiple screening methods when indicated (e.g. HPV and mobile colposcopy). Clients choose a combination of tests due to affordability, convenience, and turnaround time.

From January 1, 2016, to February 29, 2020, screening was conducted by four nurses trained in Basic Colposcopy who followed algorithms developed by the CCPTC (Figures 6, 7, 8).

Note:

A. These figures (6, 7, 8) are the algorithms used during the study period.

B. The 2021 WHO guidelines recommend using HPV DNA detection as the primary screening test rather than VIA or cytology in screening and treatment approaches among the general population of women and women living with HIV.

Upon cervical screening with the EVA System, the examination result is classified using the Rio 2011 Colposcopy Nomenclature of the IFCPC (International Federation for Cervical Pathology and Colposcopy)^17^. See Appendix 1 for a summary table. To assure the quality of assessments and results, their screening results were discussed among qualified nurse colposcopists and the lead gynaecologist of the CCPTC.

The algorithms used for screening are attached (Figures 6, 7, and 8). Women were scheduled for rescreening per the protocol when no lesion was observed on the cervix. The nurses treated women with lesions with ablation therapy (cryotherapy or thermal coagulation) if they qualified for treatment.

Women who did not qualify for ablation treatment were referred to a gynaecologist for further management, which usually involved biopsy and loop electrosurgical excision procedure (LEEP).

### Statistical analysis

We provide statistics using frequencies and percentages to describe clients screened using mobile colposcopy, their screening results, and the cervical precancer treatment modalities. STATA version 14.2 (StataCorp LLC, College Station, TX, USA) was used for the analyses. Analysis items with P <0.05 were considered statistically significant.

### Ethical approval

In line with practice at Catholic Hospital Battor, The Ethical Review Committee of the Catholic Hospital, Battor approved (CHB-ERC-002/07/19) for this review to be carried out and for it to be published (clearance ID: 0202/01/22). All patients consented to procedures and treatment.

## Results

Over the study period, a total of 904 women underwent cervical screening at the CCPTC at the Catholic Hospital, Battor, by four nurses using mobile colposcopy (EVA).

### Sociodemographic and clinical details

The social and demographic characteristics are presented in Table 1a. The average age of the women was 37.6 years (SD= 11.1), a majority (70%) were married or had a steady partner, and approximately 60% had at least one child. Almost half (49.3%) had a minimum of secondary school education, and more than 99% had never smoked. At the time of screening, 11.1% were HIV+, and 9.7% were using contraceptives. Less than a fifth (18.2%) had a history of cervical screening, and less than 1% had ever been treated for a cervical condition.

**Table 1a:** Socio-demographic characteristics of partici-pants from September 2016 to February 2020

Client Characteristic	Estimate
**Age, Mean (Standard Deviation)**	37.6 (11.1)
**Marital Status**	**% (n)**
Single	17.2 (155)
Has a steady partner	20.1 (182)
Married	50.9 (460)
Divorced	5.0 (45)
Widowed	5.6 (51)
Missing	1.2 (11)
**Number of children**	**% (n)**
0	38.6 (349)
1	17.9 (162)
2	15.9 (144)
3	10.9 (99)
4+	15.0 (136)
Missing	1.6 (14)
**Highest Level of Education**	**% (n)**
No formal education	5.4 (49)
Elementary education	6.1 (55)
Secondary education	27.1 (245)
Tertiary education	22.2 (201)
Missing	39.2 (354)
**Religious Faith**	**% (n)**
Christianity	56.6 (512)
Islam	3.7 (33)
African Traditional Religion	0.4 (4)
Missing	39.3 (355)
**Current Smoker**	**% (n)**
Yes	0.2 (2)
No	99.2 (897)
Missing	0.6 (5)
**HIV Status**	**% (n)**
Positive	11.9 (108)
Negative	29.0 (262)
Unknown Never tested	39.6 (358)
Missing	19.5 (176)
**Current Contraceptive Use**	**% (n)**
Yes	9.7 (88)
No	89.7 (811)
Missing	0.6 (5)

### Mobile colposcopy screening details

The clinical characteristics are presented in tables 1 (b) and table 2. Of the 904 women, 91.6% underwent the EVA system primary screening by mobile colposcopy.

**Table 1b:** Clinical characteristics of clients who under-went cervical screening performed by trained nurses us-ing the EVA System from September 2016 to February 2020

Client Characteristic	Estimate
**History of Cervical Screening**	**% (n)**
Yes	18.2 (151)
No	81.8 (677)
**Previous Cervical Treatment**	**% (n)**
Yes	0.9 (8)
No	98.6 (891)
Missing	0.6 (5)
**Primary Screening, % (n)**	91.6 (828)
Cervical Inspection	% (n)
Normal	96.4 (871)
Abnormal	3.6 (33)
**Vaginal Inspection**	**% (n)**
Normal	97.3 (880)
Abnormal	2.7 (24)
Adequate View with Mobile Colposcopy***, **% (n)**	99.3 (896)
**Positive EVA Findings (Among Ad-equate** **EVA), % (n)** *	14.4 (130)
Minor Change	69.2 (90)
Major Change	24.6 (32)
Leukoplakia	2.3 (3)
Suspicion for invasion/cancer	3.1 (4)
Benign lesions	0.8 (1)
Cervical Lesions**, **% (n)**	14.5 (130)
**Treated for Cervical Lesions, % (n)**	2.8 (25)
Ablation	52.0 (13)
Thermal Coagulation	44.0 (11)
Cryotherapy	8 (2)
Loop Electrosurgical Excision Procedure (LEEP)	48.1 (12)

* 2 clients with no cervix and 6 with EVA colposcopy inadequate were removed from the denominator. ^†^Based on clients who underwent primary screening using EVA.

** Clients without cervix and inadequate colposcopy findings were excluded.

*** 2 clients who had no cervix were excluded

At mobile colposcopy using the EVA system, two patients were found not to have a cervix, and 6 had an inadequate view. Thus, 99.3% (896) had an adequate view. Of those with an adequate view, 130 (14.5%) had cervical lesions. Of the clients with cervical lesions, 24 (20%) were treated. Just over half of those were treated with ablation and the rest (48.0%) were treated using LEEP.

### Screening using mobile colposcopy (EVA) in addition to other screening methods

Table 2 provides details on screening by mobile colposcopy (EVA) in conjunction with other screening methods such as cytology, VIA, the careHPV system, the AmpFire system, and the GeneXpert system. For primary screening, 739 (89.3%) were done at the CCPTC clinic at Battor. Of those who underwent primary screening at the clinic, 92 (12.4%) had cervical lesions, compared to 15 (16.9%) of those screened during outreach. Follow-up screening was done for 64 women at the clinic and 12 during outreaches. Upon follow-up at the clinic, 31.3% had cervical lesions compared to 25% among the follow-up screening clients during outreaches. Approximately a third (34.4%) of the patients were screened by mobile colposcopy (EVA) alone, 14.3% were screened with both EVA and Ampfire HPV testing system, 19.9% were screened with both EVA and careHPV, 14.6% were screened with cytology and EVA, and 14.3% were screened with EVA and GeneXpert HPV testing system.

**Table 2:** Screening, including mobile colposcopy with the EVA system from September 2016 to February 2020. Positives (EVA positives) are in brackets

	Primary Screening§		Follow up (Screen Positives) ¶		
	Clinic	Out- reach	Clinic	Out- reach	Total
**EVA Alone**	221 (35)	50 (6)	29 (9)	11 (3)	311 (53)
**AMPFIRE+EVA**	102 (21)	20 (4)	6 (2)	1 (0)	129 (27)
**CareHPV+EVA**	164 (17)	5 (0)	11 (4)	0 (0)	180 (21)
**EVA+PAP**	127 (6)	0 (0)	5 (0)	0 (0)	132 (6)
**GeneXpert+EVA**	116 (13)	2 (0)	11 (5)	0 (0)	129 (18)
**VIA+EVA**	0 (0)	11 (5)	0 (0)	0 (0)	11 (5)
**Care-** **HPV+EVA+PAP**	4 (0)	1 (0)	0 (0)	0 (0)	5 (0)
**EVA+Care-** **HPV+VIA**	2 (0)	0 (0)	0 (0)	0 (0)	2 (0)
**Gen-** **Xpert+EVA+Care-** **HPV**	1 (0)	0 (0)	0 (0)	0 (0)	1 (0)
**GeneX-** **pert+EVA+PAP**	2 (0)	0 (0)	2 (0)	0(0)	4 (0)
**Total**	739 (92)	89 (15)	64 (20)	12 (3)	904 (130)

§ Primary screening refers to those who came in for cervical screening without any prior (positive) result.

¶ The follow-ups (screen positives) are those who either came in from another facility with a positive test (through pap, VIA and HPV testing) or that had already been screened at CCPTC, were positive and returned for a follow-up test.

## Discussion

Outside low-resource settings that use Visual Inspection with Acetic acid (VIA), cervical precancer screening typically involves primary screening with cytology and HPV DNA testing and follow-up of screen positives with colposcopy. 18 Traditionally, colposcopy has been performed using a stationary colposcope.^19^ In low-resource settings, programs using cytology, HPV DNA testing, and colposcopy (particularly with traditional stationary colposcopes) are difficult to implement because of the high costs.^20^ Another major limitation to cervical cancer screening with HPV DNA testing in low-resource settings is the lack of trained colposcopists to manage screen positives.^21^, ^22^ Where there are few medical doctors, many are busy attending to other medical conditions and have not been trained to perform colposcopy. 23 To tackle these problems, many facilities in low-resource settings have adopted VIA using middle cadre staff like nurses and midwives in cervical cancer screening programs.^24^–^26^ In addition, implementing a see-and-treat screening protocol has the added advantage.

The use of mobile colposcopes has simplified cervical precancer screening.^27^,^28^ There is evidence that some mobile colposcopes perform similarly to traditional stationary colposcopes.^29^,^30^ Several studies have shown that there is no difference in performance between medical doctors and middle cadre staff like nurses in colposcopy 31 and that there is no difference in performance between doctors and nurses when using the mobile colposcope.^19^ Although many studies have demonstrated the feasibility and accuracy of portable digital colposcopes in low-resource settings, these have not been routinely used in clinical practice, particularly alongside HPV DNA testing.^32^–^34^

In our model, we trained middle cadre staff (nurses and midwives) to perform VIA and basic mobile colposcopy. Mobile colposcopy was used for primary screening, follow-up of high-risk HPV-positive cases, and patients with abnormal pap smears (ASCUS or worse). During the study period (from September 2016 to December 2019), four trained nurses performed screening using mobile colposcopy at Catholic Hospital, Battor. Eighty-nine (9.8%) of the colposcopic examinations were performed on outreaches, many in remote communities, including community-based health planning services (CHPS) compounds and health centres, where it would have been difficult to transport a stationary/traditional colposcope. This shows the possibility of decentralising cervical cancer prevention services to the doorsteps of women. The cost of colposcopy performed by trained nurses using a mobile colposcope (GHS 35 [USD 5.8]) compared to that performed using a standard stationary colposcope (GHS 120 [USD 20]) implies that more women can afford mobile colposcopy performed by trained nurses and this can reach more women, especially in remote communities.

With the dearth of medical doctors to perform colposcopy, a model using HPV DNA testing followed by colposcopy by a trained doctor would mean booking clients for follow-up colposcopy several weeks ahead. With our model using trained nurses, one could perform colposcopy in the same setting when an HPV sample was taken or much earlier than a referral to a physician. The performance of colposcopy has so far been limited to medical doctors in Ghana 35. Our experience shows that nurses supervised by an experienced gynaecologist can achieve good results using mobile colposcopy. Whereas the existing policy^35^ seeks to reduce the apparent harms of misdiagnosis when non-specialists perform screening procedures, our work has shown that in settings where gynaecologists are few, nurses can offer services in colposcopy with a gynaecologist performing a supervisory role. In our experience, some stationary colposcopes are without cameras to capture images.

This makes reviewing images during quality assurance impossible, even if a medical doctor/gynaecologist performed the colposcopy. In our opinion, the current mobile colposcopes used by our nurses give better images than some of the older stationary colposcopes with cameras. These mobile colposcopes make it possible to perform colposcopy on outreaches on CHPS compounds and also provide images that can help in quality assurance

Quality assurance is key to successfully following through with any cervical cancer screening program.^36^ Our facility uses a team care model and collaborative practice for quality assurance in cervical cancer screening through monthly quality assurance meetings in which in-house and external nurses affiliated with the CCPTC discuss cases with a specialist gynaecologist in attendance. A previous study has reported that the addition of quality assurance review by specialists using digital colposcopy increases the diagnostic value of VIA for predicting CIN 2+ lesions from 65% to 75% (P = 0.001).36 Nurses in more remote facilities can also engage with specialist gynaecologists anonymised images on the WhatsApp® platform. Colposcopic images can be uploaded to the cloud for quality assurance reviews. This allows for expert consultation with a remote expert before the patient leaves clinic^37^ and allows continuous quality improvement.^38^

Nurses can treat lesions in the same setting (“screen and treat”) or at a later date with cryotherapy or thermal coagulation. In this study, among the clients with cervical lesions, 24 (20%) were treated. Just over half of those treated with ablation, and the rest (48.0%) were treated using LEEP. Lesions unamenable to treatment were referred to a doctor for a loop electrosurgical excision procedure (LEEP). The possibility of nurses carrying out ablative treatment in remote communities reduces the number of clients lost to follow-up.

Artificial intelligence has questioned the role of training nurses and other middle cadre health workers in Basic Colposcopy for the future. The possible futuristic use of automated visual evaluation (AVE) means there may be shorter learning curves for nurses and other health workers to perform colposcopy. Artificial intelligence will help less experienced health workers arrive at a diagnosis.^39^,^40^,^41^ Follow-up and treatment of precancerous lesions of the cervix, either with ablation (cryotherapy, thermal coagulation) or with LEEP, may still require similar training as are available currently, and experienced nurses and other cadres of staff are needed to handle them in low resource settings.

The use of AVE in cervical precancer screening may make screening easier with less need for many highly trained personnel; however, trained middle cadre staff would most likely be needed as treatment of premalignant lesions of the cervix will require a review of the images and the knowledge and skills of this trained personnel. Our study had some limitations. Although our approach is expected to be utilised by middle cadre staff, our work relied on screening performed by a single cohort of nurses at a single facility. Images were taken using contemporary mobile colposcopes by nurses trained in VIA and Basic Colposcopy, and not other categories of healthcare workers.

## Conclusion

The roles of middle cadre staff in cervical cancer screening in low-income settings have largely centred around education, outreach, and creating awareness. We have demonstrated that middle cadre staff, like trained nurses, can perform colposcopy in a cervical cancer screening programme in low-resource settings using mobile colposcopes, ensuring that women can be screened and followed up in the clinic setting and communities. This addresses the problem of triaging screen positives with colposcopy, a major concern in screening programmes using HPV testing due to the lack of doctor colposcopists, especially in low-resource settings.
